# Improving Multiscale Fuzzy Entropy Robustness in EEG-Based Alzheimer’s Disease Detection via Amplitude Transformation

**DOI:** 10.3390/s24237794

**Published:** 2024-12-05

**Authors:** Pasquale Arpaia, Maria Cacciapuoti, Andrea Cataldo, Sabatina Criscuolo, Egidio De Benedetto, Antonio Masciullo, Marisa Pesola, Raissa Schiavoni

**Affiliations:** 1 Department of Electrical Engineering and Information Technology, University of Naples Federico II, 80125 Naples, Italy; pasquale.arpaia@unina.it (P.A.); sabatina.criscuolo@unina.it (S.C.); marisa.pesola@unina.it (M.P.); 2 Department of Engineering for Innovation, University of Salento, 73100 Lecce, Italyandrea.cataldo@unisalento.it (A.C.); antonio.masciullo@unisalento.it (A.M.); raissa.schiavoni@unisalento.it (R.S.)

**Keywords:** electroencephalography, biomedical signal processing, multiscale fuzzy entropy, Alzheimer’s disease, complexity, measurements, measures

## Abstract

This study investigates the effectiveness of amplitude transformation in enhancing the performance and robustness of Multiscale Fuzzy Entropy for Alzheimer’s disease detection using electroencephalography signals. Multiscale Fuzzy Entropy is a complexity measure particularly sensitive to intra- and inter-subject variations in signal amplitude, as well as the selection of key parameters such as embedding dimension (*m*) and similarity criterion (*r*), which often result in inconsistent outcomes when applied to multivariate data, such as electroencephalography signals. To address these challenges and to generalize the possibility of adopting Multiscale Fuzzy Entropy as a diagnostic tool for Alzheimer’s disease, this research explores amplitude transformation preprocessing on electroencephalography signals in Multiscale Fuzzy Entropy calculation across varying parameters. The statistical analysis of the obtained results demonstrates that amplitude transformation preprocessing significantly enhances Multiscale Fuzzy Entropy’s ability to detect Alzheimer’s disease, achieving higher and more consistent significant comparison percentages, with an average of 73.2% across all parameter combinations, compared with only one raw data combination exceeding 65%. Clustering analysis corroborates these findings, showing that amplitude transformation improves the differentiation between Alzheimer’s disease patients and healthy subjects. These results highlight the potential of amplitude transformation to stabilize Multiscale Fuzzy Entropy performance, making it a more reliable tool for early Alzheimer’s disease detection.

## 1. Introduction

Complexity measures have emerged as convenient tools for characterizing electroencephalographic (EEG) signals, particularly in the diagnosis of neurodegenerative diseases [1,2]. Traditional EEG analysis techniques, such as event-related potentials and time-frequency analysis, often fail to capture the dynamic complexity of brain activity [2,3,4,5]. In contrast, complexity measures, particularly those based on entropy, provide a deeper understanding of the brain’s irregularities and unpredictability, offering a more nuanced approach to early diagnosis [1,6]. Among these measures, Multiscale Fuzzy Entropy (MFE) has emerged as a powerful tool for quantifying the complexity of EEG signals across different time scales [7]. MFE is based on the concept of fuzzy entropy, which quantifies the degree of regularity in a signal through the analysis of a sliding window that identifies similar patterns [8,9]. In recent years, MFE has been applied to the study of brain activity, particularly for Alzheimer’s disease (AD) [1,7,10,11].

Nevertheless, despite its potential, there is no established method for applying MFE to EEG signals, limiting comparison between studies [12,13] and ultimately hindering the reliability of MFE as biomarker of AD. The application of MFE to EEG signals faces two main challenges: its sensitivity to parameter selection and its dependence on signal amplitude [14,15]. Indeed, MFE employs a set of tunable parameters, namely *m*, which denotes the template length or embedding dimension, and *r*, which represents the matching threshold or similarity criterion [7,11]. These parameters have a significant impact on the entropy results [14]. Additionally, MFE is inherently dependent on signal amplitude [15], especially when dealing with multivariate data such as EEG. The amplitude values of EEG signals can vary significantly between different EEG systems and across subjects, thus potentially biasing the calculated distances on embedded vectors toward data with larger amplitude ranges [15,16]. Addressing these issues is crucial to standardizing MFE as a reliable biomarker for AD. Thus, this study introduces an amplitude transformation (AT) preprocessing approach to mitigate amplitude variability and improve MFE robustness across various parameter combinations. Thus, based on the hypothesis that AT preprocessing can improve the performance and robustness of MFE, the aim is to establish AT preprocessing as a key methodological enhancement for AD detection based on MFE. To this purpose, this work investigates the effectiveness of AT preprocessing by applying MFE across varying *m* and *r* parameters. Specifically, this study explores the potential of min–max amplitude normalization to enhance the performance and robustness of MFE for AD detection. MFE was assessed across 12 combinations of *m* and *r* on raw and amplitude-transformed EEG data from AD subjects and HSs. Statistical analyses, including *t*-tests (α=0.05) and Cohen’s D, were performed to assess the significance of differences between the HS and AD groups under both conditions (raw and AT data) for each MFE scale factor and EEG channel, with each parameter combination. Additionally, a clustering analysis using *k*-means was conducted to evaluate the clustering capability based on MFE values. The findings demonstrate that AT preprocessing significantly enhances the performance and robustness of MFE, making it less sensitive to the chosen parameters *m* and *r* and more reliable in distinguishing between HSs and subjects with AD. By effectively addressing these challenges, this paves the way for the development of more accurate diagnostic tools for Alzheimer’s disease.

The paper is organized as follows. Section 2 reports an overview of related works. Section 3 describes the theoretical background of MFE. Section 4 details the proposed method, including the dataset description, amplitude transformation strategy, MFE calculation, and used data analysis approach. Section 5 reports the obtained results, comparing raw data and amplitude-transformed data, highlighting the impact of preprocessing on the stability and robustness of MFE. Finally, the conclusions and future work are outlined in Section 6.

## 2. Related Works

The MFE approach has been employed to investigate brain function, particularly in relation to AD [1,7,10,11]. AD is the most common form of dementia, and it severely impairs cognitive and behavioral functions leading to significant neurological deterioration and affecting daily life activities [17]. Hence, early and reliable diagnosis is crucial for effective disease management and treatment. The presence of the ApoE ϵ4 allele, a known genetic risk factor for AD, has also been shown to modify EEG complexity patterns in different brain regions, particularly within the temporal lobes [18,19]. In this context, MFE has been demonstrated as suitable in differentiating between healthy subjects (HSs) and those with AD [7,10,11,20]. Table 1 provides a summary of key works, their contributions, and limitations.

Despite its potential, there is no established method for applying MFE to EEG signals, limiting comparison between studies [12,13] and ultimately hindering the reliability of MFE as a biomarker for AD. In particular, the choice of specific parameters and the amplitude of EEG signals are among the factors that influence the performance of MFE [14,15]. MFE employs tunable parameters, namely *m* (template length or embedding dimension) and *r* (matching threshold or similarity criterion) [7,11], which significantly impact the entropy results [14]. Moreover, MFE is inherently dependent on signal amplitude, especially when dealing with multivariate data such as EEG [15]. In particular, the amplitude values of EEG signals can vary significantly between different EEG systems and across subjects, thus potentially biasing the calculated distances on embedded vectors toward data with larger amplitude ranges [15,16]. For all these reasons, it is crucial to gain an understanding of the sensitivity and impact of these factors, i.e., parameters and signal amplitude, to ensure the effective application of MFE in AD detection.

Regarding parameter selection, although the literature often suggests default parameter values (m=2 and r=0.20) [22], these values are not necessarily optimal in every context. Consequently, using suboptimal parameters may result in the distortion and alteration of the obtained results [14]. This issue has been highlighted in numerous studies that have attempted to identify the best parameter configuration for specific contexts [14,23,24,25,26]. For instance, in [14], various combinations of *m* and *r* were explored to optimize MFE calculations for distinguishing between AD and HSs. Other studies have focused on evaluating electrocardiogram (ECG) signals, highlighting the need for even greater attention to parameter selection in EEG analysis [23,24,25,26]. On this basis, instead of continuously searching for the optimal parameter combination for each scenario, it would be more beneficial to develop a strategy for improving the performance of MFE by making it independent of parameter selection.

As aforementioned, the effectiveness of MFE is further complicated by the inherent variability in EEG signal amplitude, which can differ significantly both within and between subjects [27]. Typically, EEG signals are preprocessed to remove artifacts, filtering in the bands of interest, and division into epochs [28,29], neglecting the intra- and inter-subjects EEG signal amplitude variations that can lead to biased results in MFE calculation [21].

To address this dual challenge—sensitivity to parameter selection and variability in EEG signal amplitude— this work proposes an AT-preprocessing approach for mitigating the impact of parameter sensitivity and amplitude variability on MFE calculations for AD detection.

## 3. Theoretical Background

The multiscale entropy analysis method enables the complexity of a time series to be evaluated by estimating the information content across a range of temporal scales. These techniques aim to quantify the degree of regularity or chaos in a time series by analysing the signal through a sliding window that searches for similar patterns. MFE has emerged as an advanced method, evolving from traditional entropy-based techniques such as multiscale approximate entropy and multiscale approximate sample [10,30,31,32]. In contrast to the latter, which depends on binary matching (1 or 0), which can be unsuitable for highly variable biological signals, MFE leverages the theory of fuzzy logic, which embraces the concept of partial truth. According to this logic, the matching of a signal segment to a set of similar patterns is assessed using a continuous function known as the membership function. It assigns real values from 0 (indicating no match) to 1 (indicating a complete match) to quantify a certain degree of similarity among patterns throughout the entire EEG time series. Then, the total contribution of all values between 0 and 1 computed for each template will represent a measure of the complexity of the brain waveforms.

Formally, given a univariate time series x[k] of *N* samples,

(1) x[k]={x[i]:1≤i≤N},

a vector of *m* consecutive samples is considered, as follows:

(2) $$X_{i}^{m}=\left\{x\left[i\right],x\left[i+1\right],\cdots ,x\left[i+m-1\right]\right\}-x_{0}\left[i\right],$$

where *i* is the starting time point of the generic pattern and $x_{0}\left[i\right]$ is the mean value of all *m*-selected samples. More specifically, *m* represents the embedding dimension, i.e., the length of sequences to be compared.

Then, given $X_{j}^{m}$ as a shifted version of $X_{i}^{m}$, a similarity degree $D_{ij}^{m}$ of $X_{j}^{m}$ to $X_{i}^{m}$ is calculated as

(3) $$D_{ij}^{m}=\exp\left(\frac{-{\left(d_{ij}^{m}\right)}^{n}}{r}\right),$$

where $d_{ij}^{m}$ is the maximum absolute difference between the two vectors and an exponential function is used as a fuzzy membership function [33]. The smoothness of the fuzzy function is adjusted by *r*, defined arbitrarily. More specifically, *r* is known as the similarity criterion or matching threshold, and determines the sensitivity of the entropy calculation to differences between elements in the system. This parameter *r*, multiplied by the standard deviation of the data sequence, defines the tolerance interval within which two elements can be considered similar [14].

The mean similarity for all patterns of length *m* is computed as

(4) $$\phi^{m}=\frac{1}{N-m}\sum_{i=1}^{N-m}\left(\frac{1}{N-m-1}\sum_{j\neq i,j=1}^{N-m-1}D_{ij}^{m}\right).$$

Then, the mean $\phi^{m+1}$ is calculated for $X_{i}^{m+1}$. Finally, fuzzy entropy (FE) can be estimated as

(5) $$FE\left(m,n,r\right)=-\ln\left(\frac{\phi^{m+1}}{\phi^{m}}\right)$$

Thus, the fuzzy entropy represents the conditional probability that the patterns observed for *m* samples are the same for (m+1) samples.

Based on this formalism, MFE extends this by averaging FE across multiple scales:

(6) $$MFE\left(m,n,r,s\right)=\frac{1}{S}\sum_{s=1}^{S}FE^{\left(s\right)}\left(m,n,r\right)$$

where *S* is the scale factor. In this way, MFE allows the complexity of brain processes to be estimated over a time interval [10].

From these equations, it can be observed how the fuzzy entropy and its multiscale variation (MFE) are affected by signal amplitude and the parameters *m* and *r*. In more detail, increasing *m* allows for a more comprehensive reconstruction of the dynamic process, capturing more intricate details of the signal’s behavior. However, this also requires a significant number of sampling points ($10^{m}$–$20^{m}$), which can be difficult to achieve with physiological data [11]. Therefore, selecting the appropriate value of *m* is essential for balancing the depth of analysis with the practical limitations of the data. On the other hand, the parameter *r* defines the similarity criterion, establishing an interval within which two elements can be considered similar. Importantly, *r* is multiplied by the standard deviation of the signal, directly linking it to the signal’s amplitude. This relationship introduces a sensitivity to amplitude variations: signals with higher amplitudes result in a larger distances metric $d_{ij}^{m}$ between sequences, potentially increasing the entropy values. If *r* is set too low, the conditional probability estimates may become inadequate, while a high *r* value could lead to a loss of detailed signal information [11]. As such, the selection of *r* must carefully account for the signal amplitude, as it significantly affects the resulting MFE values and, consequently, the interpretation of the signal’s complexity and irregularity [15]. Based on these considerations, it is essential to develop a strategy to enhance the performance of MFE and increase its robustness against parameter variations.

## 4. Materials and Method

**Figure 1:**
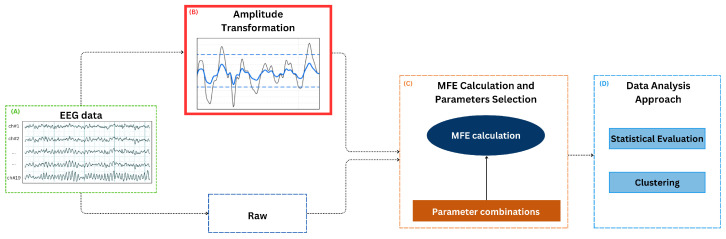
Workflow: (**A**) the process begins with data acquisition. The same EEG data are subjected to two parallel processes: the raw EEG data are analyzed to establish a baseline for comparison; (**B**) the amplitude transformation (AT) preprocessing is applied. (**C**) For each scenario (raw and AT data), Multiscale Fuzzy Entropy (MFE) is then calculated for various parameter combinations. (**D**) The data are analyzed via statistical evaluation and clustering for both amplitude-transformed and raw data. Finally, the results from these evaluations are compared to assess the robustness of the MFE calculations at parameters *m* and *r* in each scenario (raw and AT).

This section outlines the methodology proposed in this study. It begins with a description of the EEG data used for validating the method, followed by a description of the developed amplitude transformation preprocessing, which is pivotal to enhancing the robustness and reliability of MFE analysis. Then, the MFE calculation on both raw and amplitude-transformed data across various parameter combinations is reported. Finally, the data analysis methodology, including statistical tests and clustering techniques, is presented. Figure 1 shows a visual representation of the workflow.

### 4.1. EEG Data Description

The EEG data used in this study belong to two publicly available EEG datasets: the Temple University Hospital (TUH) EEG Corpus [34] and the Chung-Ang University Hospital EEG (CAUEEG) [35]. Each EEG recording is accompanied by a detailed report containing the diagnosis made by specialized neurologists. Signals were re-sampled at a frequency of 200 Hz, and the channel position used in the recordings followed the international 10-20 system, encompassing 19 channels: FP1, FP2, F3, F4, C3, C4, P3, P4, O1, O2, F7, F8, T3, T4, T5, T6, FZ, CZ, and PZ. Both datasets employed linked earlobe referencing (A1 and A2), with the ground electrode positioned at FPz. The datasets were balanced with respect to age and sex of the subjects, ensuring comprehensive and unbiased data. In particular, 17 subjects with AD and 17 HSs were selected from the TUH EEG dataset, and 13 subjects with AD and 13 HSs were selected from the CAUEG dataset. This resulted in a total of 30 AD subjects and 30 HSs, providing a dataset of 60 subjects for analysis.

For each subject, 51 s of clean EEG signals in closed-eye resting state condition was considered, as the minimum continuous closed-eye segments of EEG signals available in the two datasets. All EEG data were bandpass filtered between 0.5 Hz and 45 Hz using a finite impulse response (FIR) filter with an order equal to the number of samples in a 3 s window. Thus, the first 3 s related to the transient were excluded from each EEG signal; in this way, 48 s of filtered EEG were obtained for each subject. The EEG data were then divided into epochs of 3 s without overlap, resulting in a total of 16 epochs for each subject.

### 4.2. Amplitude Transformation

Considering the inherent dependency of MFE on signal amplitude, this study developed an AT-preprocessing procedure to investigate its effectiveness in enhancing the robustness and reliability of MFE analysis. This strategy was developed with the specific objective of mitigating the effects of intra- and inter-subject amplitude variations caused by a range of factors, such as instrumentation. By applying min–max normalization, the EEG data were scaled to a common range, minimizing inter- and intra-subject variability and preserving the proportional significance of amplitude variations. In addition, this method not only ensured more consistent results but also enhanced the robustness of MFE, making it less sensitive to the chosen parameters and more reliable in distinguishing between HSs and AD.

To implement this approach, the AT was applied to each EEG epoch as follows:

(7) $$x_{AT,i}=\frac{x_{i}-x_{min}}{x_{max}-x_{min}}\;\cdot \;\left(A_{max}-A_{min}\right)+A_{min}$$

where $x_{AT,i}$ is the amplitude-transformed signal, *i* is the *i*-th channel, $A_{max}$ and $A_{min}$ are the output amplitude-transformed ranges (from −5 to 5), empirically chosen to guarantee signal symmetry and a standard deviation of approximately 1, thereby maintaining the relative importance of each data point. For each subject, $x_{max}$ and $x_{min}$ were determined by first identifying the maximum and minimum values for each epoch. Then, the median of the maximum values and the median of the minimum values across all epochs were calculated, resulting in a single maximum value and a single minimum value per subject. A global AT approach was chosen, meaning that $x_{max}$ and $x_{min}$ were obtained, taking into account all channels simultaneously, and maintaining the amplitude ratio between channels.

### 4.3. MFE Calculation and Parameters Selection

MFE was evaluated considering raw and amplitude-transformed EEG data. In the initial stage of the analysis, the same EEG data were subjected to two parallel processes. Firstly, as reported in Figure 1, the raw EEG data (A) were analyzed to establish a baseline for comparison. Then, as shown in Figure 1B, the AT procedure was applied to each EEG signal. For each scenario (raw and AT data), the MFE was computed epoch-wise for each channel, considering the different combinations of *m* and *r* (Figure 1C). A range of scale factors from 1 to 20 was selected to effectively distinguish between AD and HSs, as indicated in prior studies [7,20]. Consequently, for each epoch, a total of 20 entropy values was yielded for each channel. These values were then averaged across epochs for each subject, resulting in 20 entropy values per channel.

More specifically, MFE was calculated for a range of combinations of its two principal parameters, *m*, which represents the embedding dimension, and *r*, which is the similarity criterion, for both the raw and amplitude-transformed datasets. As previously stated, the value of *m* determines the length of the sequences to be compared, with larger values enabling a more detailed reconstruction of the dynamic process. However, this requires a substantial number of sampling points, which can be challenging with physiological data [11]. The parameter *r* defines the threshold for the similarity between elements, balancing the accuracy of the likelihood against the potential loss of signal information [11].

Both parameters are crucial for the performance of MFE, and thus in analyzing EEG data and distinguishing between AD and HSs. As stated in Section 1, the objective of this study was to enhance the robustness of MFE with regard to the amplitude of EEG signals and parameter selection through the AT procedure. To investigate this, 12 different combinations of *m* and *r* were considered. The chosen combinations are listed in Table 2. In more detail, *m* was varied between 2, 3, and 4, while r=0.10, r=0.15, r=0.20, and r=0.25 times the standard deviation of EEG data were used. These ranges were chosen to capture a wide spectrum of potential influences on the entropy calculations, allowing for a thorough analysis of MFE’s robustness and effectiveness [14].

### 4.4. Data Analysis Approach

The data analysis approach involves a systematic examination of MFE applied to both raw and preprocessed EEG data with the AT procedure, using various parameter combinations. The analysis aims to analyze the correlation between signal amplitude and MFE parameters in distinguishing between HSs and AD. Specifically, the objective is to investigate the potential of AT preprocessing in enhancing the performance and robustness of MFE. As shown in Figure 1D, after the MFE calculation, statistical and cluster analyses were employed to assess the impact of amplitude transformation on MFE. The statistical analysis was used to evaluate the discriminatory power of MFE in each scenario (raw and AT with different parameter combinations). In more detail, the resulting MFE values from different parameter settings were statistically analyzed at each scale factor to identify the optimal parameter combinations for distinguishing between the two groups in the raw data and in AT data. Subsequently, clustering analysis was employed to further assess the performance of MFE in discriminating AD and HSs. By clustering the data, this study aimed to identify distinct groupings that corresponded to HS and AD patients. Clustering provided a complementary perspective to statistical analysis, helping to visualize the separation between the groups and offering a more intuitive understanding of the data structure. More specifically, the methods used in the analysis were as follows:

1. Statistical Evaluation
  - The *t-test*: Conducted to compare the entropy values between HSs and AD patients, determining the statistical significance of the differences observed. In particular, a two-tailed *t*-test with significance level α=0.05 was employed for all comparisons, as no specific direction of the differences between the HS and AD groups was hypothesized. This approach ensured that any significant difference, regardless of direction, was detected. To ensure the validity of the *t*-test, the assumptions of normality and homogeneity of variances were verified prior to analysis. Normality was assessed using the Lilliefors test, and homogeneity of variances was evaluated using Levene’s test. These assumptions were verified for each combination of 20 scale factors, 19 channels, and the parameters *r* and *m*, resulting in a comprehensive evaluation. The results indicated that normality was satisfied in 75.92% of the combinations and homogeneity of variances in 81.51%. Although not all combinations met these assumptions, the *t*-test was robust for sample sizes N≥30 as justified by the central limit theorem. This analysis was conducted at each scale factor. A percentage of significant comparisons (PSCs) was calculated by dividing the number of comparisons with *p*-values less than α by the total number of comparisons (channels multiplied by scale factors), then multiplying by 100, as shown in the following formula:
    (8) $$\mathrm{PSC}=\left(\frac{\sum_{i=1}^{N}1\left(p_{i}<\alpha \right)}{N}\right)\times 100$$
    where $1p_{i}<\alpha$ is an indicator function that equals 1 if $p_{i}<\alpha$, and 0 otherwise. *N* is the total number of comparisons equal to $N_{sf}\times N_{ch}$ where $N_{sf}$ is the number of scale factors and $N_{ch}$ is the number of channels.
  - Cohen’s D: Calculated to measure the effect size, providing insight into the magnitude of the differences between the groups. It is worth noting that while the *t*-test indicates whether the differences between groups are statistically significant, it does not convey the practical significance of these differences. For these reasons, Cohen’s D was adopted since it addresses this by quantifying the effect size, which helps with understanding the real-world relevance of the findings. Typically, an effect size of 0.2 is considered small, 0.5 is medium, and 0.8 is large, indicating the practical significance of the observed differences. This effect size measurement was also conducted at each scale factor using the following formula:
    (9) $$d=\frac{{\bar{x}}_{HS}-{\bar{x}}_{AD}}{s_{p}}$$
    where ${\bar{x}}_{HS}$ and ${\bar{x}}_{AD}$ are the sample means of the two groups and $s_{p}$ is the pooled standard deviation.
    (10) $$s_{p}=\sqrt{\frac{\left(N_{HS}-1\right)s_{HS}^{2}+\left(N_{AD}-1\right)s_{AD}^{2}}{N_{HS}+n_{AD}-2}}$$
    where $s_{HS}$ and $s_{AD}$ are the sample standard deviations of the HS and AD groups, respectively, and $N_{HS}$ and $N_{AD}$ are the sample sizes of the two groups. To obtain an overall evaluation of the effect size, the mean of the Cohen’s D values across scale factors and channels was calculated, according to the following formula:
    (11) $$d_{mean}=\frac{1}{N_{ch}\;\cdot \;N_{sf}}\sum_{i=1}^{N_{ch}}\sum_{j=1}^{N_{sf}}d_{ij}$$
    where $d_{i,j}$ is Cohen’s D value for channel *i* (ranging from 1 to 19) and the scale factor *j* (ranging from 1 to 20).
2. Clustering
  The *k-means* clustering approach [36] was applied to group the EEG data based on MFE values, with the goal of identifying distinct clusters corresponding to HSs and patients with AD. The number of clusters *k* was set to 2, representing the two groups (HSs and AD). The feature matrix was constructed with data from 30 HSs and 30 AD patients, resulting in a total of 60 subjects. For each subject, the mean of the MFE values across the 20 scale factors was calculated for each of the 19 channels, yielding a single entropy value per channel. This resulted in a feature matrix of dimensions 60×19, where each row represented a subject and each column represented a specific MFE feature for one of the 19 channels. The clustering performance was evaluated using the V-measure [37], Adjusted Mutual Information (AMI) [38], and Adjusted Rand Index (ARI) [39] metrics. These methods were used in order to assess the quality of the clusters generated by *k-means*, ensuring that they accurately reflected the underlying patterns in the data. Specifically, the V-measure, ranging between 0 and 1, enables the evaluation of both the homogeneity and completeness of clusters. It quantifies the degree of similarity between the samples within a cluster and the extent to which similar samples are grouped together by the clustering algorithm. The AMI is a measure of the degree of overlap between two cluster assignments. It is a value between 0 and 1, with 0 indicating complete independence and 1 indicating complete similarity. Finally, ARI is a metric that quantifies the degree of similarity between two data partitions: the clustering results and the true labels. It assumes values within the range of [−1,1]. Negative values signify the independence of splits, whereas positive values indicate that these splits are consistent.

## 5. Experimental Results and Discussion

In this section, the obtained results across various parameter combinations for both raw and processed EEG data are presented, and detailed statistical and clustering analyses are provided to evaluate the correlation between the signal amplitude and parameters of MFE in distinguishing between HSs and AD.

### 5.1. Preliminary Entropy Analysis

**Figure 2:**
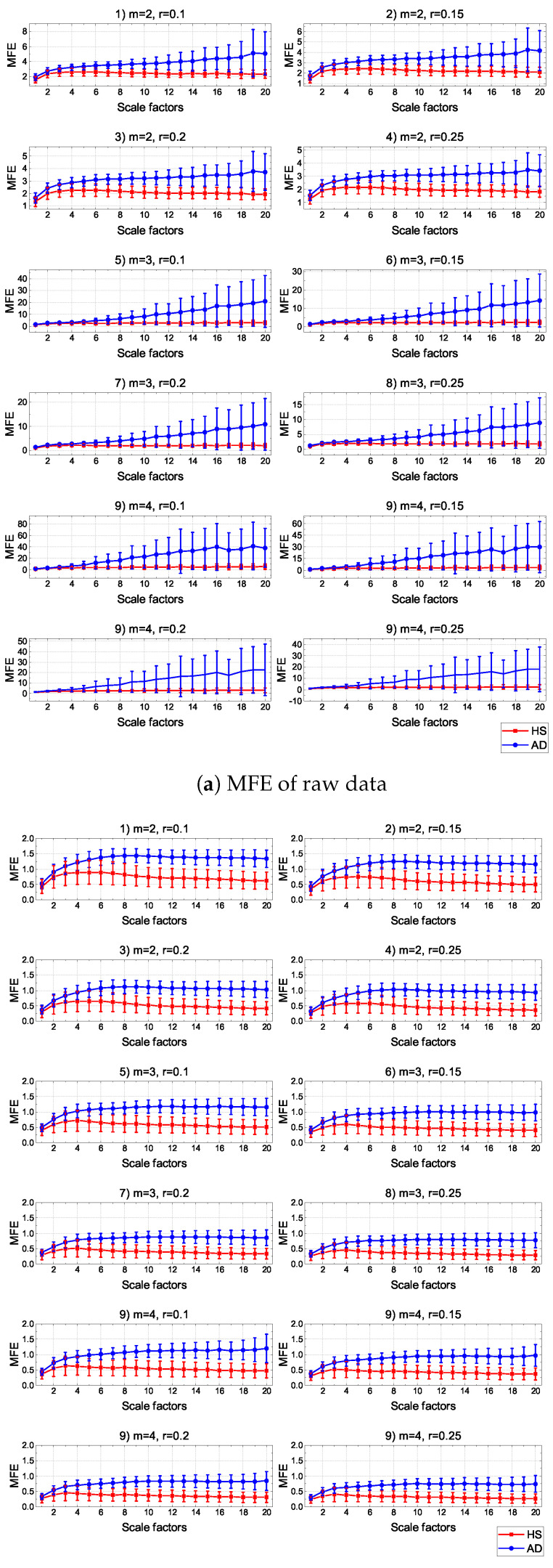
Comparison of MFE on 20 scale factors for raw (**a**) and processed data with AT procedure (**b**) by using CZ electrode.

Figure 2 shows the MFE results for different scale factors across the established parameter combinations, using the CZ channel as an example. Specifically, for each group, the mean across the subjects and the associated standard deviation are reported for each scale factor. The visual representation of entropy variation demonstrates the differences in discrimination capability between the two groups (HS and AD) for both raw and AT-processed data. In more detail, in the case of raw data, it was evident that a certain separability between the groups was noticeable only for combinations 1 to 4. For the other combinations, there was a flattening of the entropy values for HS and an excessive increase in the standard deviation bars for AD, making it difficult to separate the groups effectively. Consequently, these results are not reliable for use as reference data for the development of diagnostic tools. The observed variability in raw data can be attributed to intrinsic EEG irregularities in AD, which are linked to disrupted neural connectivity and increased complexity in brain activity. Furthermore, structural and functional brain differences, particularly in regions affected by AD such as the temporal and parietal lobes, contribute to the heterogeneity of EEG signals in AD subjects. In contrast, for AT-processed data, the entropy trends for AD and HSs remain consistently distinguishable across all parameter combinations. This improved separability highlights the impact of AT preprocessing, which normalizes amplitude-related variability across datasets and subjects. By reducing the influence of raw signal amplitude on MFE calculations, AT enhances the robustness and reliability of the entropy measures, ensuring that the observed differences more accurately reflect the intrinsic signal complexity rather than artifacts of signal preprocessing.

This preliminary observation suggests that AT preprocessing addresses challenges related to signal amplitude and parameter sensitivity, thereby enhancing the discriminatory power of MFE in clinical diagnostics.

These results were further quantified through the following statistical and clustering analyses.

### 5.2. Statistical Analysis Results

**Figure 3:**
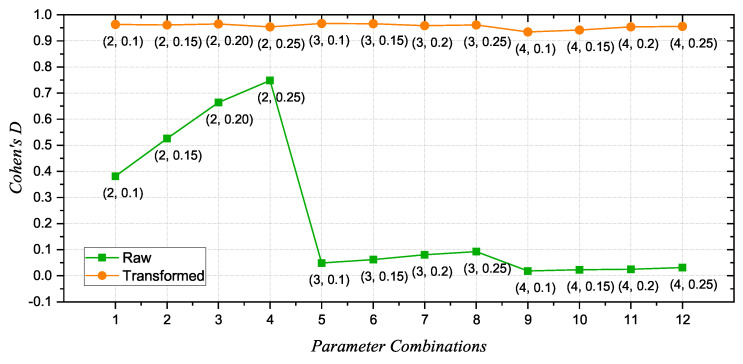
Cohen’s D values for raw and AT data across the 12 parameter combinations.

The *t*-test was applied to each scale factor and channel for each parameter combination to determine the significance of differences between the HS and AD groups. The results are summarized in Table 3, which presents the *t*-value, *p*-value, Cohen’s D, and PSCs for each combination of parameters *m* and *r*, both for raw and AT-processed data. Since the *t*-test was performed for each scale factor, channel, and parameter combination (20×19×12 tests in total), the *t*-value and *p*-value were reported as ranges to better capture the variability across all these tests. For the raw data, the *t*-values ranged from 0.00 to 9.49, while the AT-processed data exhibited a broader range, from 0.01 to 10.29. Similarly, the *p*-values spanned from $3.38\times 10^{-12}$ to $9.82\times 10^{-1}$ for raw data and from $1.53\times 10^{-14}$ to $9.49\times 10^{-1}$ for AT-processed data. These ranges reflect the significant improvement in discriminative power introduced by the AT procedure, with consistently lower *p*-value minima and higher *t*-value maxima across all parameter combinations. Additionally, Cohen’s D was calculated to measure the effect size, providing insights into the magnitude of differences between the HS and AD groups and the practical significance of the observed results. See Figure 3. For the raw data, the mean Cohen’s D values confirmed moderate discrimination for the first four combinations, with values ranging from 0.48 ± 0.10 to 0.75 ± 0.10. However, starting from the fifth combination, the mean values dropped significantly, reaching as low as 0.35 ± 0.10 for the twelfth combination (m = 4 and r = 0.25). In contrast, the AT-processed data exhibited consistently higher mean Cohen’s D values across all parameter combinations, ranging from 0.95 ± 0.07 to 0.97 ± 0.07. This stability across all combinations highlights the robustness of MFE in distinguishing between the two groups when the AT procedure is applied. To better highlight the improvement in the discrimination between the two groups and the proportion of statistically significant comparisons, particular attention should be given to the PSCs. This metric provides a more immediate and intuitive understanding of the fraction of comparisons that reach statistical significance, summarizing the overall effectiveness of the parameter combinations in distinguishing between the HS and AD groups. The PSC values clearly demonstrate that the AT procedure not only improves the robustness of the analysis but also ensures consistently higher discrimination performance across all parameter combinations. In more detail, the PSCs for the raw data were generally lower than the AT-processed data, with the fourth combination (m=2 and r=0.25) appearing to be the most effective. In particular, the first four parameter combinations showed a moderate level of separability between the HS and AD groups, with significant comparison percentages exceeding 65%. However, from the fourth combination onward, the percentages dropped drastically, reaching as low as 39.48% for m=4 and r=0.10. The overall mean PSC for the raw data was 59.30%, with a standard deviation of 9.10%. As previously discussed, the variability in PSC values for raw data can also be linked to the inherent heterogeneity of EEG signals in AD patients, driven by disrupted neural synchronization and variations in brain network connectivity. These irregularities are further amplified by the absence of preprocessing, which leaves raw signal amplitudes and noise unfiltered, leading to reduced consistency in entropy-based separability measures. These findings are also in accordance with the results of previous studies on the impact of parameters in fuzzy entropy in differentiating between AD and HSs [14].

In addition, to ensure the reported findings’ reliability, the analysis of statistical power was calculated. The calculation was based on the mean effect size represented by the mean Cohen’s D ($CD_{mean}=0.9567$), derived from the results obtained after the AT transformation, since it enhances the differences between the HS and AD groups. The standard approach for two-sample *t*-tests [40] was adopted, where the Type II error β is defined as

(12) $$\beta =P\left(t<t_{critical}-CD_{mean}\;\cdot \;\sqrt{\frac{N_{HS}\;\cdot \;N_{AD}}{N_{HS}+N_{AD}}}\right)$$

where *P* is the cumulative probability under the t-distribution, $N_{HS}$ and $N_{AD}$ are the sample sizes for the HS group and AD group, respectively, each consisting of 30 subjects, and $t_{critical}$ is the critical *t*-value determined based on a significance level of α=0.05 and degrees of freedom df=58$\left(N_{HS}+N_{AD}-2\right)$. The statistical power, calculated as 1−β, was found to be 95.31%, confirming that the sample size was sufficient to detect the observed effect size.

### 5.3. Clustering Outcomes

Table 4 summarizes the clustering results in terms of V-measure, ARI, and AMI for each parameter combination. For the raw data, the results show that the clustering performance is generally low, with the V-measure, ARI, and AMI values being relatively modest. The fourth combination (m=2,r=0.25) stands out with higher values across all three metrics, indicating that this combination provides the best separability between HS and AD subjects among the raw data. However, for the remaining combinations, the performance drops significantly, highlighting the sensitivity of MFE to parameter variations when applied to raw data. In contrast, the AT-processed data demonstrate markedly improved and more stable clustering performance across all combinations. The V-measure, ARI, and AMI values are consistently higher compared with those from the raw data, with the highest values observed for combinations 6 and 12.

**Figure 4:**
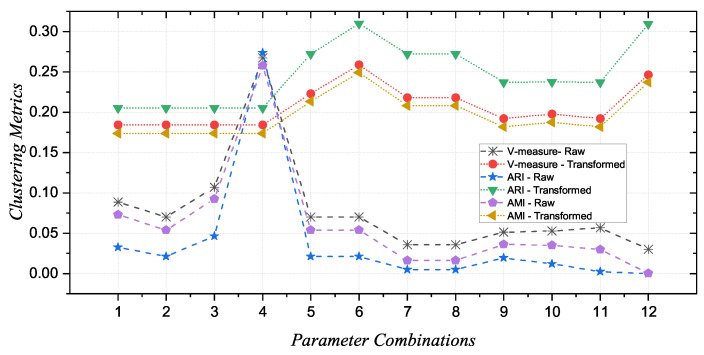
Comparison of clustering performance metrics (V-measure, ARI, AMI) for raw and transformed EEG data across 12 parameter combinations. The graph highlights the peak in performance for the fourth parameter combination in raw data and shows the consistently higher and stable performance metrics for processed data, indicating the robustness of the AT procedure.

Furthermore, these three metrics are plotted together in Figure 4 as they vary within the same range of values.

This visual representation allows for immediate and clear observation of the peak present at the fourth combination for the raw data, with the other metric values declining sharply. In contrast, the AT-processed data show a trend of consistently higher and more stable values across all three metrics, reinforcing the notion that the preprocessing step effectively stabilizes the MFE results. This stability suggests that the AT procedure enhances the robustness of MFE, making it less sensitive to the choice of parameters.

**Figure 5:**
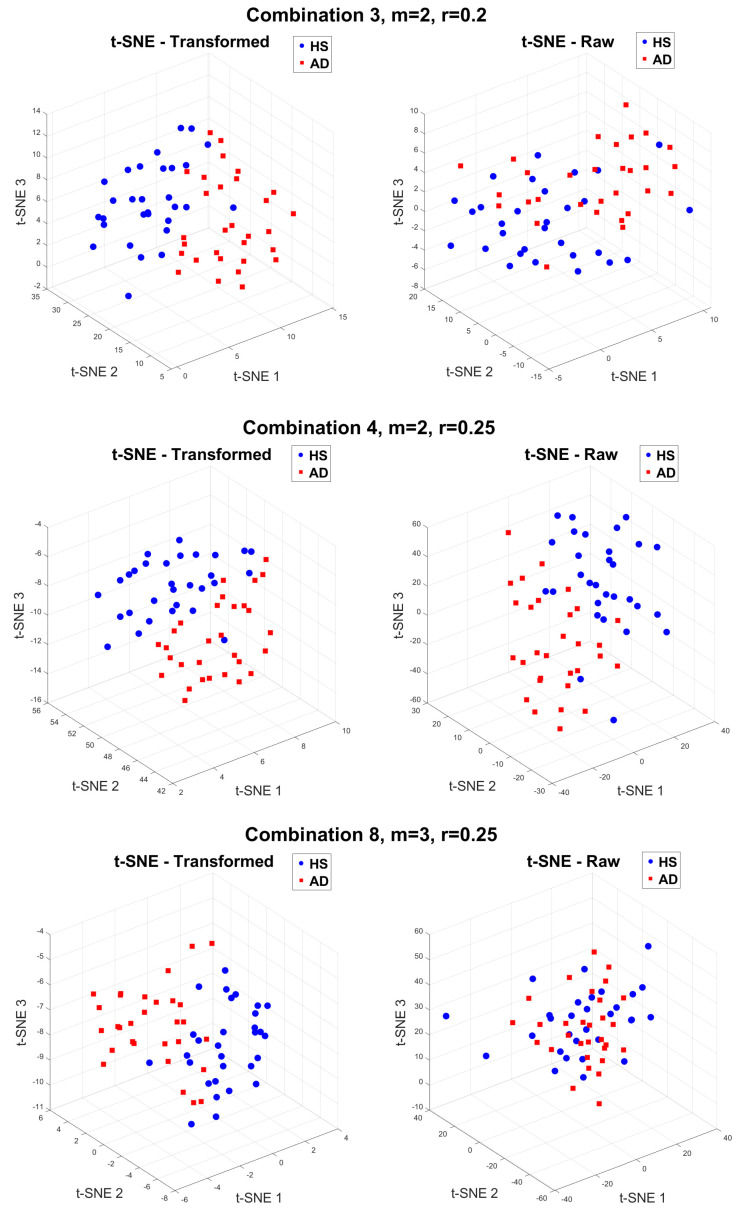
Three-dimensional t-SNE plots for clustering visualization in the case of transformed data on the left and raw data on the right. Blue circles represent HSs, while red squares indicate AD subjects.

As a further confirmation of the obtained results, Figure 5 reports the clustering of HSs and AD subjects by using the t-Distributed Stochastic Neighbour Embedding (t-SNE) technique. The t-SNE is a non-linear dimensionality reduction method that is particularly effective for visualizing high-density data by projecting them into a low-density space, typically two or three dimensions, while preserving the local structure of the data [41]. In this study, the first three principal components were extracted, and the data were plotted to observe the spatial distribution of HSs and AD subjects. For brevity, visualizations were provided for three parameter combinations, reporting transformed data on the left and raw data on the right. Blue circles represent the HSs, while red squares indicate the AD subjects. In particular, Figure 5 includes clustering visualization for the following:

- Combination 3 (m = 2, r = 0.2): default parameter settings, which serve as a basis for comparison.
- Combination 4 (m = 2, r = 0.25): the best performing for raw data, where the clustering metrics (V-measure, ARI, and AMI) have reached their maximum values.
- Combination 8 (m = 3, r = 0.25): a scenario following the best combination, where performance for the raw data decreases significantly.

### 5.4. Discussion

The results show that, without AT, the performance of MFE on raw data is highly dependent on the choice of parameters *m* and *r*. This dependency arises because the fuzzy entropy algorithm calculates distances between vectors in the signal, which are directly influenced by the amplitude of the EEG signal. As a result, a single value of *r* may not effectively capture similarities across the entire signal, as it could be too small for some parts and too large for others. This variation makes it challenging to select an optimal *r* that works consistently across the signal, leading to a less robust FuzzyEn that is more sensitive to the data’s amplitude. This may lead to an incorrect assessment of the true complexity of the EEG signals. Consequently, selecting optimal parameters for *m* and *r* becomes challenging, and the reliability of fuzzy entropy as a complexity measure is compromised. The application of AT preprocessing significantly mitigated these issues by normalizing the EEG signal amplitudes. This normalization ensures that the distances calculated between the vectors are consistent, regardless of the amplitude variations across different sections of the signal. As a result, MFE’s sensitivity to the parameters *m* and *r* was significantly reduced, as demonstrated by the more stable and robust performance across all parameter combinations in the AT-preprocessed data. Additionally, AT preprocessing may minimize variability introduced by differences in EEG acquisition systems, such as variations in reference electrode placements or montage configurations, which could otherwise affect signal amplitudes. This standardization enhances the method’s robustness and comparability across datasets, making it less susceptible to artifacts introduced by acquisition conditions and ensuring generalization.

The effectiveness of the AT procedure was confirmed by the statistical and clustering analysis. Without AT, the MFE algorithm shows significant variability, particularly in its sensitivity to different parameter combinations, with only few combinations exceeding a PCS of 65%. This variability poses a challenge for reliable discrimination between the HS and AD groups, as the raw data often resulted in inconsistent outcomes. In contrast, the AT-processed data exhibited significantly higher and more stable performances across all parameter combinations. The percentage of significant comparisons consistently exceeded 70%, indicating that AT effectively eliminates the sensitivity of MFE to parameter variations. Finally, in the clustering analysis, amplitude-transformed data highlighted clearer separability between the HS and AD groups.

This approach reduces the need for fine-tuning parameters and simplifies the implementation process, which is particularly beneficial in clinical settings where efficiency and simplicity are crucial. In addition, maintaining high accuracy irrespective of the chosen parameters increases the generalizability of the method across different datasets and experimental conditions. Furthermore, the improved reliability of predictions ensures that the results are less likely to be artifacts of specific parameter settings and more likely to reflect the true complexity of the EEG signals.

## 6. Conclusions

This study highlights the crucial role of amplitude transformation in improving the robustness and reliability of MFE for EEG-based discrimination between HSs and patients with AD across various parameter combination. The obtained results demonstrate that the inherent dependency of MFE on data amplitude and its sensitivity to parameter variations were effectively mitigated by the AT procedure. Moreover, the statistical and clustering results confirm that not only does AT improve the robustness of MFE but it also enhances its ability to differentiate between the two groups, making it a valuable preprocessing step in EEG analysis for neurodegenerative disease detection.

The proposed approach reduces the necessity for fine-tuning parameters and simplifies the implementation process, which is particularly advantageous in clinical settings where efficiency and simplicity are paramount. Moreover, the enhanced reliability of predictions ensures that the results are less likely to be artifacts of specific parameter settings and more accurately reflect the true complexity of EEG signals.

Further investigations have been planned to explore additional aspects. For example, while the AT procedure in this study is based on min–max normalization, alternative normalization techniques will be examined to enhance the analysis of MFE. Additionally, future studies will expand the sample size by including a larger cohort and considering potential comorbidities. To complement these efforts, the integration of Current Source Density (CSD) analysis will also be explored [42]. By improving spatial resolution and isolating local neuronal activity, CSD could provide a refined input for MFE analysis, potentially unveiling new insights into the interplay between spatial and temporal neural dynamics. This comprehensive approach will pave the way for a more robust evaluation of the method’s efficacy and its future validation in real clinical settings. 

## Figures and Tables

**Table 1:** Multiscale Fuzzy Entropy analysis of EEG signals for detecting Alzheimer’s disease (AD) in existing research.

Author(s)	Year	Methodology	Data Used	Key Findings	Limitations
Simons et al. [14]	2018	Fuzzy Entropy (FuzEn)	EEG from 22 subjects (AD and HSs)	FuzEn outperformed other entropy metrics in distinguishing AD from HSs, achieving 86.36% accuracy.	Small sample size; heavy dependence on input parameters for entropy calculation.
Azami et al. [11]	2019	Fuzzy entropy with various membership functions (MFs)	Synthetic clinical datasets	Gaussian MF yielded the best performance for long signals; exponential MF for the short ones.	Limited comparability across methods without defuzzification; no focus on AD.
Su et al. [10]	2021	MFE and Phase Locking Value (PLV)	EEG from 49 subjects (AD and HSs)	Combined MFE and PLV achieved 83.34% classification accuracy.	Focused on prefrontal regions; limited generalizability to other brain areas.
Cataldo et al. [7]	2024	Multiscale Fuzzy Entropy (MFE)	Public EEG dataset	MFE showed a trend inversion in complexity across frequency bands for AD patients.	Requires more robust validation for clinical use; dataset size could be larger.
Arpaia et al. [21]	2024	MFE	Two public EEG datasets	Emphasis on the preprocessing phases. The importance of data normalization, in improving the effectiveness of clustering algorithms for AD identification.	The method has been tested on two public datasets, but the data are still scarce for application in the clinical field.

**Table 2:** Combinations of parameters *r* and *m* for MFE.

Combination	*m*	*r*
1	2	0.10
2	2	0.15
3	2	0.20
4	2	0.25
5	3	0.10
6	3	0.15
7	3	0.20
8	3	0.25
9	4	0.10
10	4	0.15
11	4	0.20
12	4	0.25

**Table 3:** Comparison of *t*-value, *p*-value, Cohen’s D, and PSCs for raw and transformed data (degrees of freedom df=58).

Comb	*t*-Value (Range)		*p*-Value (Range)		Cohen’s D (Mean ± std)		PSC (%)	
	Raw	Transformed	Raw	Transformed	Raw	Transformed	Raw	Transformed
1	[0.02, 8.75]	[0.06, 10.19]	[3.38 $\times 10^{-12}$, 9.82 $\times 10^{-1}$]	[1.53 $\times 10^{-14}$, 9.49 $\times 10^{-1}$]	0.48 ± 0.10	0.96 ± 0.08	66.84	72.37
2	[0.05, 9.17]	[0.01, 10.21]	[6.88 $\times 10^{-13}$, 9.63 $\times 10^{-1}$]	[1.43 $\times 10^{-13}$, 9.90 $\times 10^{-1}$]	0.51 ± 0.11	0.96 ± 0.06	69.74	72.89
3	[0.06, 9.33]	[0.00, 10.27]	[3.84 $\times 10^{-13}$, 9.55 $\times 10^{-1}$]	[1.14 $\times 10^{-12}$, 9.99 $\times 10^{-1}$]	0.52 ± 0.11	0.96 ± 0.05	71.84	72.89
4	[0.03, 9.49]	[0.00, 10.29]	[2.06 $\times 10^{-13}$, 9.72 $\times 10^{-1}$]	[1.07 $\times 10^{-12}$, 9.85 $\times 10^{-1}$]	0.55 ± 0.10	0.96 ± 0.06	72.63	73.15
5	[0.00, 5.49]	[0.02, 10.05]	[9.30 $\times 10^{-7}$, 9.99 $\times 10^{-1}$]	[2.56 $\times 10^{-14}$, 9.87 $\times 10^{-1}$]	0.36 ± 0.12	0.97 ± 0.07	54.47	73.42
6	[0.01, 5.45]	[0.05, 10.04]	[1.08 $\times 10^{-6}$, 9.95 $\times 10^{-1}$]	[2.71 $\times 10^{-14}$, 9.63 $\times 10^{-1}$]	0.36 ± 0.12	0.96 ± 0.07	55.79	73.42
7	[0.00, 5.83]	[0.09, 9.99]	[2.59 $\times 10^{-6}$, 9.93 $\times 10^{-1}$]	[2.03 $\times 10^{-14}$, 9.99 $\times 10^{-1}$]	0.37 ± 0.11	0.96 ± 0.08	56.84	72.89
8	[0.00, 6.21]	[0.00, 9.96]	[6.12 $\times 10^{-8}$, 9.97 $\times 10^{-1}$]	[3.79 $\times 10^{-14}$, 9.99 $\times 10^{-1}$]	0.37 ± 0.11	0.96 ± 0.07	57.11	72.63
9	[0.02, 5.54]	[0.02, 9.86]	[7.65 $\times 10^{-7}$, 9.87 $\times 10^{-1}$]	[5.14 $\times 10^{-14}$, 9.82 $\times 10^{-1}$]	0.36 ± 0.11	0.93 ± 0.06	39.47	72.63
10	[0.02, 5.51]	[0.01, 10.04]	[8.60 $\times 10^{-7}$, 9.86 $\times 10^{-1}$]	[2.68 $\times 10^{-14}$, 9.91 $\times 10^{-1}$]	0.36 ± 0.11	0.95 ± 0.07	52.89	73.42
11	[0.02, 5.48]	[0.00, 10.13]	[9.71 $\times 10^{-9}$, 9.84 $\times 10^{-1}$]	[1.89 $\times 10^{-14}$, 9.99 $\times 10^{-1}$]	0.36 ± 0.11	0.95 ± 0.07	56.84	73.95
12	[0.01, 5.45]	[0.01, 10.17]	[1.09 $\times 10^{-6}$, 9.94 $\times 10^{-1}$]	[1.64 $\times 10^{-14}$, 9.93 $\times 10^{-1}$]	0.35 ± 0.10	0.96 ± 0.07	57.11	74.47

**Table 4:** Comparison of clustering metrics (V-measure, ARI, AMI) for raw and transformed data, with mean and standard deviation (std) in bold.

Comb	V-Measure		ARI		AMI	
	Raw	Transformed	Raw	Transformed	Raw	Transformed
1	0.08	0.18	0.03	0.21	0.07	0.17
2	0.07	0.18	0.02	0.21	0.05	0.17
3	0.11	0.18	0.05	0.21	0.09	0.17
4	0.27	0.18	0.27	0.21	0.26	0.17
5	0.07	0.22	0.02	0.27	0.05	0.21
6	0.07	0.26	0.02	0.31	0.05	0.25
7	0.04	0.22	0.01	0.23	0.02	0.21
8	0.04	0.22	0.01	0.27	0.01	0.21
9	0.05	0.19	0.02	0.24	0.04	0.18
10	0.05	0.19	0.01	0.24	0.03	0.19
11	0.06	0.19	0.01	0.24	0.03	0.18
12	0.03	0.25	0.00	0.31	0.00	0.24
mean ± std	**0.06 ± 0.02**	**0.21 ± 0.03**	**0.02 ± 0.02**	**0.025 ± 0.04**	**0.07 ± 0.07**	**0.19 ± 0.02**

## Data Availability

The original contributions presented in this study are included in the article. Further inquiries can be directed to the corresponding author.
